# Stable carbon and nitrogen isotopes in kukui (*Aleurites moluccanus*) endocarp along rainfall and elevation gradients: Archaeological implications

**DOI:** 10.1371/journal.pone.0204654

**Published:** 2018-10-16

**Authors:** Noa Kekuewa Lincoln, Mark D. McCoy, Thegn N. Ladefoged

**Affiliations:** 1 Department of Tropical Plants and Soil Sciences, University of Hawai‘i, Honolulu, Hawaii, United States of America; 2 Department of Anthropology, Southern Methodist University, Dallas, Texas, United States of America; 3 Anthropology, University of Auckland, Auckland, New Zealand; 4 Te Pūnaha Matatini, New Zealand Centre of Research Excellence, Auckland, New Zealand; University of Otago, NEW ZEALAND

## Abstract

Stable carbon and nitrogen isotopes are often used to make inferences of past environments and social patterns. We analyze δ ^13^C and δ ^15^N values in contemporary kukui (*Aleurites moluccanus*) endocarp to examine the effects of site environment. Results from across environmental transects on Hawai‘i Island show strong patterns for both stable isotopes. For δ ^13^C a robust linear relationship with elevation is exhibited, strengthened by the inclusion of rainfall. This relationship breaks down at a minimum threshold of annual rainfall, possible relating to physiological responses to drought. For δ ^15^N, the only significant relationship observed pertains to substrate age. The endocarp from kukui is one of the most readily identified plant remains in the Pacific archaeological records and is often targeted for radiocarbon dating. We discuss the potential implications of our results regarding ancient climate, inferred diets, and habitat composition.

## Introduction

Stable isotopes, particularly those of carbon and nitrogen, are often used to make inferences on past environments and social patterns, from inferring diets of ancient humans [1, 2] and animals [3, 4], to examining changing weather and climate [5–7] to reconstructing past habitat composition [8] and processes [9]. Carbon and nitrogen stable isotope analysis is a well-established technique for reconstructing diet from human or animal tissue, made possible by consistent and predictable isotopic fractionation throughout food webs. δ ^13^C values can be used to determine the proportions of C_3_ and C_4_ foods consumed, as differential ^13^C fractionation during photosynthesis generates distinct values for C_3_ versus C_4_ plants, which are then passed reliably up the food chain [10]. Nitrogen stable isotope ratios can be used to estimate trophic position within a food web, as δ ^15^N values undergo a ~3–5‰ stepwise enrichment between trophic levels [11–13]. Unfortunately within such studies, less attention is paid to the local environmental drivers of stable isotope ratios, which can cause significant δ ^13^C and δ ^15^N variation between individuals within even a single species. Identifying and controlling for such variation is critical to accurate archaeological interpretations of food webs and diet.

The stable carbon isotope ratio of terrestrial plant material varies based on many environmental and physiological characteristics related to photosynthetic pathways and leaf gas exchange, due to a discrimination of the heavier ^13^C isotope in several pathways. For C_3_ plants, the fractionation processes are primarily controlled by the ratio of intercellular to ambient CO_2_ concentrations, which is affected by both environmental and response factors [14]. Prominently, as atmospheric pressure decreases with increasing altitude the vapor pressure difference of the leaf and atmosphere decreases and a greater discrimination of the heavier isotopes occurs. However, this discrimination effect can be skewed by plant physiological responses that can occur on short time frames, such as leaf adjustments for stomatal conductance [15], or as permanent adaptations, such as higher internal resistance to CO_2_ diffusion with leaf thickness [16]. The responses are affected by aspects of the environment that necessarily vary with altitude (e.g., solar radiation, temperature, atmospheric pressure) and by environmental aspects that vary independently of altitude (e.g., rainfall, humidity, wind speed).

Environmental and physiological factors control the stable nitrogen isotope ratios of terrestrial plants. Physiologically, the dominant mechanism is atmospheric nitrogen fixation, although less well-understood mechanisms of fractionation during uptake are reported. Environmental factors mainly drive differences in the isotope ratios of available nitrogen in the soil, thereby affecting the value found in plants. Globally, a small but significant relationship exists between δ ^15^N and precipitation. In Hawai‘i, a positive relationship between δ ^15^N and soil age was reported, thought to represent accumulated losses of ^15^N-depleted nitrogen later in soil development [17].

Broad, highly organized elevational and rainfall gradients exist in Hawai‘i over short geographical ranges. This feature of the Hawaiian environment makes it highly conducive to the study environmental forcing on natural processes of pedogenesis, physiology, and ecological function [18]. It also allows for unique opportunities for sourcing of elemental sources in lithic material and soils [19] and biological materials [20] based on different isotope signatures from the different environments. We examined patterns of δ ^13^C and δ ^15^N in the endocarp of the candlenut tree (kukui, *Aleurites moluccanus*), a commonly preserved botanical remain recovered in archaeological investigation in Hawai‘i, to evaluate the use of isotope analysis in paleoclimatology, past food webs, and landscapes in Hawaiian archaeology.

## Materials and methods

**Fig 1:**
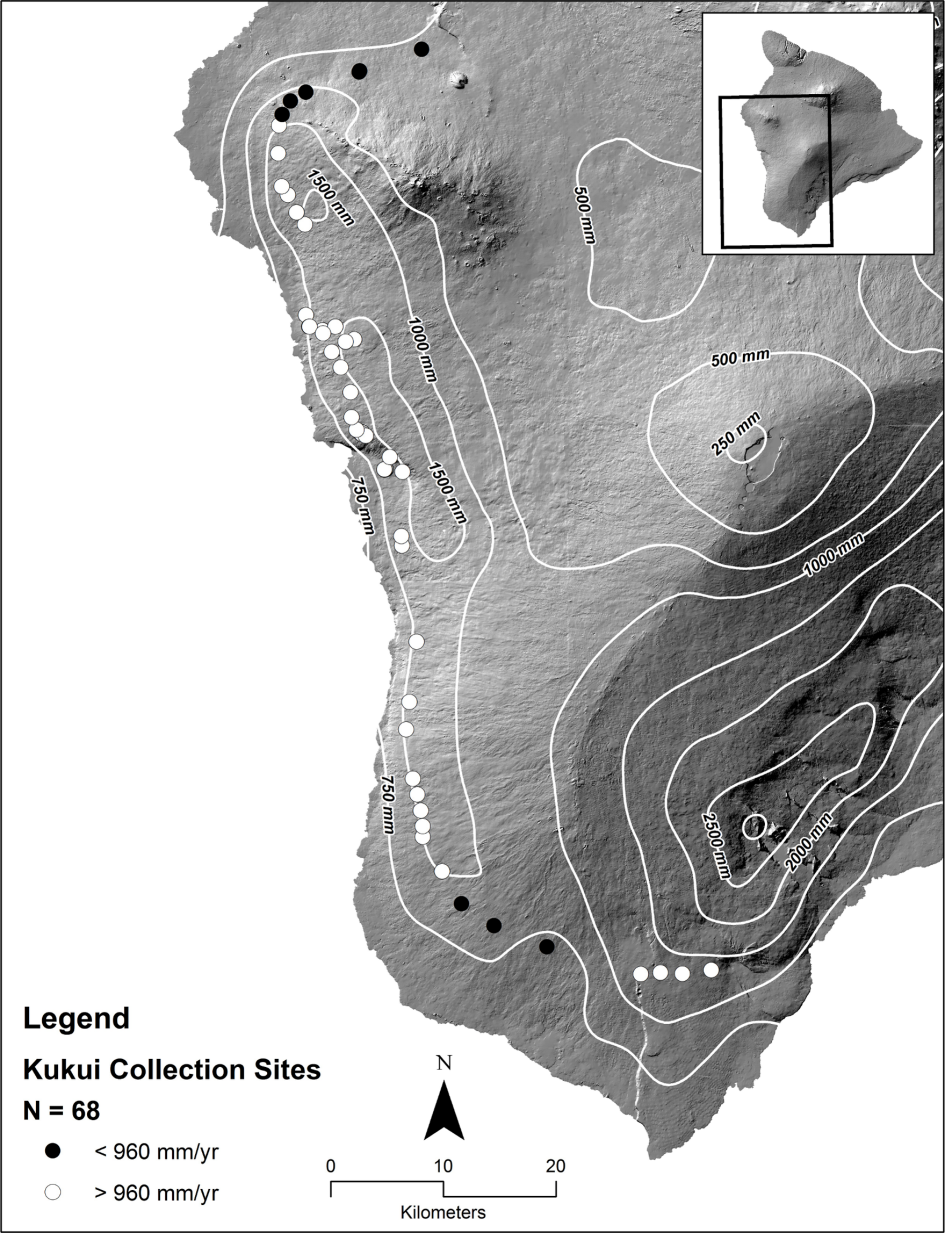
Map of sampling locations. Each point represents collection site representing multiple trees; black dots indicate areas that receive less an annual rainfall of less than 970 mm/yr.

From July to December 2015 we collected 506 ripe kukui fruit from 71 sites along the western and southern parts of Hawai‘i Island, Hawai‘i (Fig 1). Site coordinates were recorded with GPS, and locations were later used to acquire environmental parameters using available GIS layers from the Hawai‘i Statewide GIS Program (http://planning.hawaii.gov/gis/). Sites were accessed on private lands with permission, and did not involve or impact endangered species. Multiple collection patterns were made. One transect was conducted that spanned an elevational gradient from ~100 to 800 m, and a second transect stayed within a relatively narrow band of elevation between 400 to 600 m but spanned a rainfall gradient of 550 to 1550 mm/yr. Two localized areas were sampled more intensively to examine the effect of tree age and height. Additional selective sampling occurred to examine the effects of soil age at a similar elevation and rainfall.

Five to eight fruit were harvested from each tree, collected from canopy branches, and three trees were sampled for each treatment. Fruits were husked to reveal the nuts, which were cleaned with steel wire brushes and cracked open. Endocarp was retained and dried in a low temp oven for 144 hours. Dried endocarp were buffed with a fine steel brush to remove any excess residue, pulverized in a ball mill, and weighed samples were encapsulated in tin. Each sample represents a single nut; each site represents multiple trees and multiple nuts from each tree. Values from each sample were used for the analysis; due to low variability samples were ultimately bundled by site, with variation at both the tree and site level reported.

Samples were sent to UC Davis Stable Isotope facility (http://stableisotopefacility.ucdavis.edu) for analysis of total carbon and nitrogen, δ ^13^C, and δ ^15^N. The laboratory applies a continuous flow Isotope Ratio Mass Spectrometer, using an Elementar Vario EL Cube or Micro Cube elemental analyzer (Elementar Analysensysteme GmbH, Hanau, Germany) interfaced to a PDZ Europa 20–20 isotope ratio mass spectrometer (Sercon Ltd., Cheshire, UK). Two different calibrated laboratory standards and intersperses replicates are applied to ensure accuracy, with final values corrected and expressed relative to international standards VPDB (Vienna PeeDee Belemnite) and Air for carbon and nitrogen respectively. The long-term standard deviation is 0.2 ‰ for δ^13^C and 0.3 ‰ for δ ^15^N.

## Results

Results showed high levels of ^13^C discrimination, with δ ^13^C values ranging from -20.8 to -29.5 ‰. (S1 Table). Discrimination levels were examined against a range of environmental parameters known to have direct and indirect effects on ^13^C discrimination in plants, some of which resulted in highly significant, but weak, relationships when examined across all samples (Table 1). The parameters that show a significant relationship to δ ^13^C are all strongly correlated to elevation in Hawai‘i (radiation, humidity, vapor pressure, temperature) [21, 22]. The two primary parameters of interest, elevation (i.e., location on the landscape) and rainfall (i.e., climate) showed r^2^ values of 0.309 and 0.125, respectively, when viewed across all samples; to examine the interaction of these two variable, δ ^13^C values were also considered against the ratio of elevation to rainfall.

**Table 1:** Correlation of δ ^13^C values to climate parameters.

Climate Parameter	δ ^13^C(‰) Response (r^2^)		
	<960 mm/yr	>960 mm/yr	All Samples
Elevation	——	0.814**	0.309**
Rainfall	——	——	0.125**
Diffuse Radiation	——	0.633**	0.359**
Soil Moisture	——	——	0.067*
Relative Humidity	——	0.744**	0.363*
Vapor Pressure Differential	——	0.736**	0.376**
Surface Temperature	——	0.492**	0.318**
Aridity	——	0.125**	0.241**
Soil Age Class	——	——	——
Elevation:Rainfall	——	0.849**	——

Correlation as represented by r^2^ values; samples represented subsets above and below the identified threshold in annual rainfall of 970 mm/yr, and for all samples;

* indicates p values <0.05,

** indicates p values <0.01,

——indicates no significant relationship.

**Fig 2:**
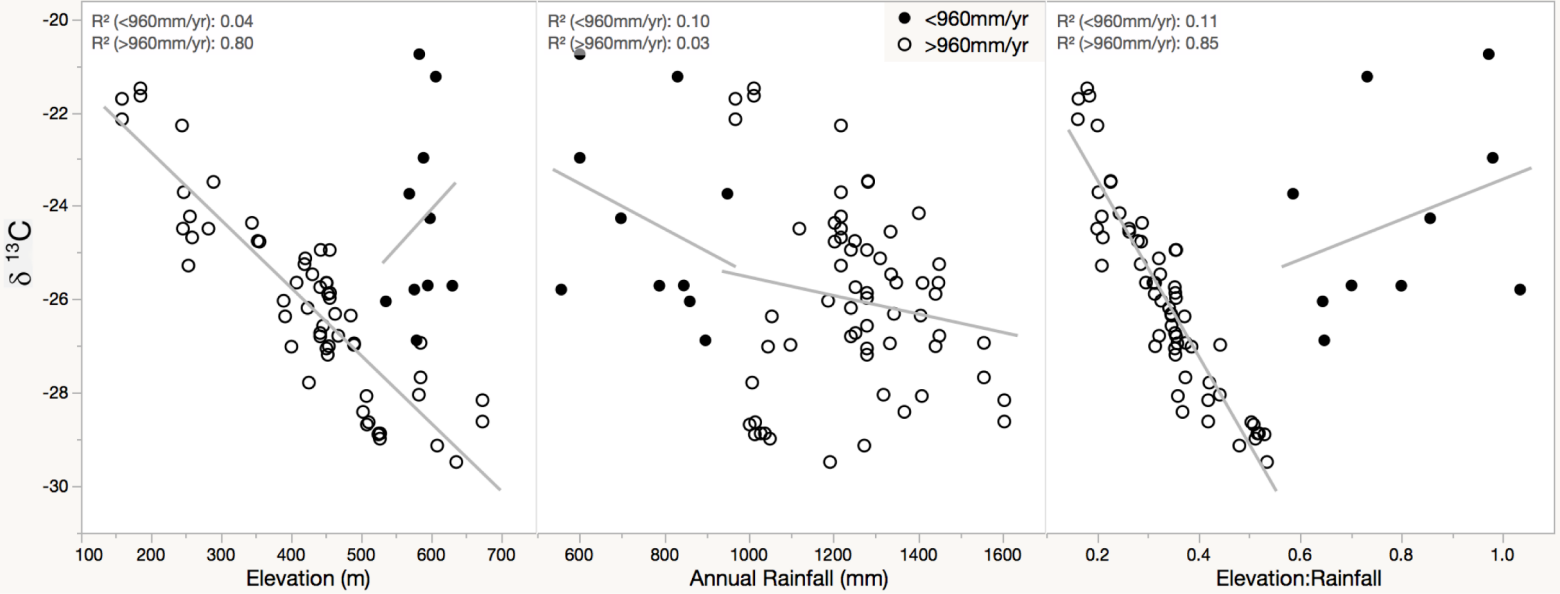
δ ^13^C values in kukui endocarp. Response of δ ^13^C to elevation, rainfall, and the ratio of elevation:rainfall; solid dots represent sites that receive an annual rainfall of less than 970 mm/yr of rainfall, and hollow dots represent sites that receive an annual rainfall of more than 970 mm/yr.

While the r^2^ values were relatively weak when considered for all samples, a clear visual pattern was evident when examing the plots, which exhibited a strong linear pattern with a handful of outliers. This pattern was exceptionally apparent in plotting δ ^13^C against elevation:rainfall; here, the outliers were easily categorized as all the sample points representing an elevation:rainfall ratio of greater than 0.55 (Fig 2). The high ratio was driven by rainfall levels, with the outliers representing all samples with an annual rainfall of lower than 970 mm/yr. When considering only samples with an annual rainfall above 970 mm/yr, the linear relationship of all parameters increased (Table 1), with strong linear relationships of δ ^13^C with elevation (r^2^ 0.814) and elevation:rainfall (r^2^ 0.849); the ten outlier sites (less than 970 mm/yr) showed no significant relationships to any of the environmental parameters (Table 1).

Surprisingly, no effects of tree size or substrate age were seen, despite samples varying considerably in tree age, from juvenile trees in the early stages of fruiting to massive trees known to be over 80 years old, and in substrate age, from exposed young lava rock to soils over one meter in depth. Pseudo-replicate analyses showed excellent agreement, with the average variation of δ ^13^C less than 0.05 ‰, indicating that our method was accurate and replicable. Multiple fruits analyzed from a single tree showed average variation in δ ^13^C of less than 0.5 ‰, while multiple trees analyzed within a single site showed a similar variation in δ ^13^C of less than 0.6 ‰. The similar levels of variation within and between trees indicate that the variability between individual fruit drives most of the variation at the site level, with differences at the tree level adding negligible variation.

**Fig 3:**
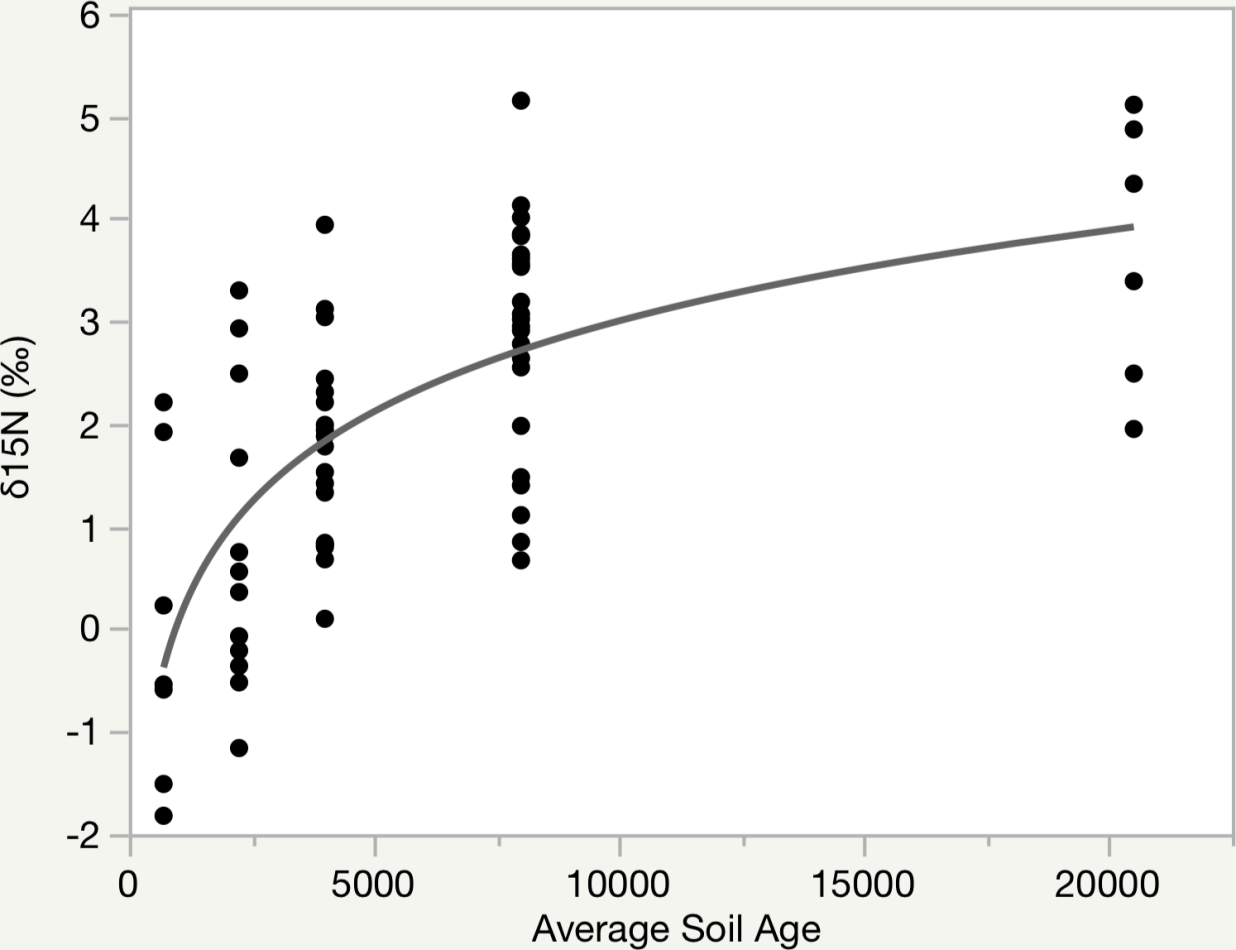
δ ^15^N values in kukui endocarp. Response in δ ^15^N to soil age by average substrate age of the age class exhibiting a log-linear relationship.

Discrimination in ^15^N was evident, with δ ^15^N values ranging from -1.8 to 5.2 ‰ (S1 Table). When applying a similar analysis of climate and environmental drivers to the δ ^15^N values, the only significant relationship was in relation to soil age class (r^2^ 0.465, p<0.001; Fig 3A). No effects of any climatic values were significant nor visually apparent, and the rainfall threshold observed for δ ^13^C values did not affect δ ^15^N values. When applying an average age of the soil substrate, rather than an age class, the δ ^15^N exhibited a log-linear relationship with soil age (r^2^ 0.483, p<0.001; Fig 3B). All trees were unmanaged (fallow) trees that we do not think differed in management inputs.

## Discussion

Both carbon and nitrogen isotopes in kukui endocarp showed strong levels of discrimination that could be significantly related to climate and environmental factors. Carbon isotopes expressed a threshold relationship with rainfall, above which δ ^13^C values were predominantly explained by elevation, which relates strongly to other significant climate parameters such as vapor pressure differential, humidity, temperature, and radiation. Nitrogen isotope values only exhibited a relationship with the underlying substrate age.

In all trees at all sites that receive an annual rainfall of more than 970 mm/yr, a strong linear pattern between δ ^13^C and elevation was evident, with a ratio of elevation:rainfall explaining approximately 84% of the variation seen in δ ^13^C values. This relationship may be exceptionally high in Hawai‘i, where multiple environmental characteristics are correlated with elevation. Some of these necessarily covary with elevation, such as vapor pressure and temperature, and will hold across any site globally. Other parameters, such as relative humidity and rainfall, do not necessarily vary with elevation and may make Hawai‘i particularly amenable to the relationship. Sites that receive an annual rainfall of less than 970 mm/yr showed no patterns with any of the environmental parameters examined; the sample size was admittedly small, but the highly insignificant relationship appears to indicate that no relationship would exist even with more robust sampling. The low rainfall sites are all significantly less discriminatory than wet sites, which would correspond to a higher ratio (lower differential) of partial pressure between the leaf and the atmosphere and a higher stomatal conductance [15]. The threshold pattern observed was extremely strong, and may indicate the point at which kukui becomes water stressed, potentially deviating from its standard stomatal operations. Similarly, this may represent the point at which the landscape shifts from a positive to negative water balance. The 970 mm/yr threshold in rainfall identified in this study closely corresponds to shifts in the agricultural applications of the early Hawaiians, closely mirroring the cutoff values for agricultural intensification in Kohala [23, 24], and the point at which arboricultural developments (the kalu‘ulu zone) gave way to resource cultivation (the kula zone) in Kona [17, 25, 26].

Conversely, δ ^15^N showed no relationship to any climate parameters nor elevation, however a significant positive relationship was seen with soil age. This agrees with previous findings in Hawai‘i, with the theoretical driver being preferential loss of ^14^N from soils over time [27]. The log-linear relationship observed with soil age seems to indicate that this relationship would stabilize over time and that the effect of soil age on old soils would be negligible. Globally, patterns between ^15^N in soil have been shown to negatively correlate with rainfall, and, moreover, that foliar ^15^N further discriminates soil values in a negative relationship with rainfall [28, 29]. Along a very broad climiate sequence on an older substrate in Hawai‘i, δ ^15^N was shown to vary considerably, from +4 ‰ at high rainfall to +14 ‰ at the drier end, likely driven by different sources and dynamics of nitrogen cycling [30]. Despite the predicted relationship with rainfall, we saw no significant correlation; this is likely due to the very young soils we sampled, meaning the cumulative effect of rainfall over time was still insignificant. Emerging work in Hawai‘i shows a very strong negative relationship between ^15^N in soils and rainfall on older substrates in Kohala [31].

These patterns in the stable isotope values of a commonly preserved botanical feature in archaeological samples have potential implications for improving paleo-reconstruction of climate, landscape, and diet. Such applications must take caution for natural variations in isotopic levels, as well as anthropogenic-caused variations. Most critically, for carbon isotopes, the Suess effect, or increased atmospheric CO_2_ concentrations due to anthropogenic emissions, must be accounted for when considering archaeological samples [32]. There are also natural variations in atmospheric CO_2_ and carbon isotope concentrations that may vary with global biomass, particularly on the time scale of the ice ages [33]. Archaeological samples change in the isotopic ratios during diagenesis, and prehistoric samples may differ from their modern counterparts by as much as 8% and 21% for carbon and nitrogen, respectively [34]. Additional changes to carbon and nitrogen isotope ratios have been shown to occur if archaeological samples are charred, although more studies are needed to better quantify such effects [35].

We show there are three factors at play in the interpretation of C and N values from ancient kukui samples recovered from archaeological deposits: first a binary division between water-stress and not water stress, then a two-factor driver of elevation and annual rainfall levels. While limiting the extent of paleo-recreation, these relationships offer opportunities for interpretation under certain conditions. For example, it is possible that the relative frequencies of water-stressed samples found at the same location may change over time due to the influnce of ENSO; an adgent of drought in the Hawaiian Islands. In addition, the strong linear relationship of δ ^13^C to elevation:rainfall suggests that, once the δ ^13^C was appropriately corrected for, rainfall could be determined based on elevation of the location where the samples were recovered. This requires controling for the transport of kukui, and in many cases that will be impossible. Nonetheless, this study shows that, in some circumstances, outlier δ ^15^N values may be indicative of kukui nutshell having been transported from a different environment that does not match the soil age of the local environment.

This robust analysis of stable isotope composition in a model system supports previous research that elevation, and in particular its relationship to vapor pressure differential, is a core driver to δ ^13^C values [14, 36]. Therefore, particular care is required when extrapolating co-variants to altitude, such as temperature, as a driver. For instance, extrapolation of ancient temperature has been conducted using modern samples along an elevation gradient for calibration (e.g., [37]). This assumes that temperature or rainfall is the dominant driver of the changes as they vary along an elevation gradient, ignoring the other environmental drivers of carbon discrimination that necessarily vary with altitude; in such cases, the temperature effect is overestimated, with the elevational effect on pressure differential and humidity likely playing the dominant effect. Furthermore, other changes in paleoclimate over time, such as rainfall, also necessarily impact values and therefore interpretations of other dependent variables, such as temperature or food webs. It is possible that, although the physiological mechanisms of ^13^C are reasonably well understood, this information has not adequately penetrated the communities outside of plant physiology. Case in point, a previous review of isotope drivers in trophic ecology attributed the decrease in δ ^13^C with altitude solely to the changing composition of C3 and C4 plants, giving no consideration to the climatic drivers [38]. With the growing application of isotopes in archaeological research, we strongly suggest that a paper outlining cosiderations of isotopes in archaeological research be produced as a disciplinary standard.

The modern values we derive may also be useful in more accurate interpretations of landscape composition and ancient diets that use carbon and nitrogen isotopes, often in mixing models, to draw conclusions about past ecosystems [39, 40]. Many times these models use generic numbers for classes of foods, such as C3 plants, fungi, etc. While more robust studies conduct sampling of the modern environment to generate the figures used in their models, a more robust understanding of climate and environmental drivers to stable isotopes can be applied to improve the mixing algorithms for such paleo-reconstructions. In particular, as it pertains to Pacific Islands, it may be that the elevation of excavations can undergo a correction model to allow for more precise mixing of broad categories, such as C3 and C4 plant composition. This method could be particularly useful when using localized indicators, such as short-ranged scavengers or local-level sediment collection [41].

## Conclusion

This paper provides a robust assessment of stable carbon and nitrogen isotopes from within a model ecological system of a commonly preserved botanical sample in archaeological settings. We provide some discussion of the implications the results have for common, and current, interpretations extrapolated within paleo-studies. δ ^13^C in kukui endocarp shows a strong linear relationship with a ratio of elevation to rainfall, exhibiting a threshold break in the relationship at an annual rainfall of 970 mm/yr. δ ^15^N values show a strong relationship to soil age, but this particular study system is unique in its young substrates, and likely overshadows the environmental drivers that are evident in older substrates. Overall, the use of δ ^13^C in kukui endocarp is a potential tool to assess paleo-climate. Given that carbon isotopes are regularly investigated on botanical material in archaeological investigations, and that kukui endocarp is highly preserved in most Hawaiian archaeological sites, this information is relevant for sourcing and inferred movement of prehistoric materials in Hawai‘i.

## Supporting information

S1 Table Average data for the 71 sites sampled for this investigation. Specific location, relevant environmental details, and carbon and nitrogen total and isotopic data included.
(XLSX)
